# Revealing the Enhancement and Degradation Mechanisms Affecting the Performance of Carbonate Precipitation in EICP Process

**DOI:** 10.3389/fbioe.2021.750258

**Published:** 2021-11-23

**Authors:** Wenle Hu, Wen-Chieh Cheng, Shaojie Wen, Ke Yuan

**Affiliations:** ^1^ School of Civil Engineering, Xi’an University of Architecture and Technology, Xi’an, China; ^2^ Shaanxi Key Laboratory of Geotechnical and Underground Space Engineering (XAUAT), Xi’an, China

**Keywords:** enzyme-induced carbonate precipitation, hijacking mechanism, magnesium ions, ammonium ions, test tube experiment

## Abstract

Given that acid-rich rainfall can cause serious damage to heritage buildings in NW China and subsequently accelerate their aging problem, countermeasures to protect their integrity and also to preserve the continuity of Chinese culture are in pressing need. Enzyme-induced carbonate precipitation (EICP) that modifies the mechanical properties of the soil through enhancing the interparticle bonds by the precipitated crystals and the formation of other carbonate minerals is under a spotlight in recent years. EICP is considered as an alternative to the microbial-induced carbonate precipitation (MICP) because cultivating soil microbes are considered to be challenging in field applications. This study conducts a series of test tube experiments to reproduce the ordinary EICP process, and the produced carbonate precipitation is compared with that of the modified EICP process subjected to the effect of higher MgCl_2_, NH_4_Cl, and CaCl_2_ concentrations, respectively. The modified EICP, subjected to the effect of higher MgCl_2_ concentrations, performs the best with the highest carbonate precipitation. The enhancement mechanism of carbonate precipitation is well interpreted through elevating the activity of urease enzyme by introducing the magnesium ions. Furthermore, the degradation of carbonate precipitation presents when subjected to the effect of higher NH_4_Cl concentration. The decreasing activity of urease enzyme and the reverse EICP process play a leading role in degrading the carbonate precipitation. Moreover, when subjected to the effect of higher CaCl_2_ concentrations, the slower rate of urea hydrolysis and the decreasing activity of urease enzyme are primarily responsible for forming the “hijacking” phenomenon of carbonate precipitation. The findings of this study explore the potential use of the EICP technology for the protection of heritage buildings in NW China.

## Introduction

In recent years, microbial-induced carbonate precipitation (MICP) and enzyme-induced carbonate precipitation (EICP) involving biomineralization have been studied. They aim not only to improve the mechanical and thermal behaviors of problematic soils (e.g., calcareous sand) (Neupane et al., 2013; Carmona et al., 2016; Putra et al., 2016; Almajed et al., 2018; Li et al., 2020; Xu et al., 2020; Li et al., 2020; Xiao et al., 2020; Bai et al., 2021a; Bai et al., 2021b; Bai et al., 2021c; Wu et al., 2021) but also to achieve the durability strengthening of concrete (Achal et al., 2010; Tittelboom et al., 2010; Achal et al., 2013; Liu et al., 2020a; Sun et al., 2021). MICP requires the existence of ureolytic bacteria, urea, and calcium-rich solution to drive the MICP biochemical reaction (Bang et al., 2001; Bu et al., 2018; Li et al., 2017; Wen et al., 2019a), which leads to ammonium and carbonate ion, and the produced carbonate ions react with calcium ions to precipitate as calcium carbonate crystals (Hammes, 2003; Hammes et al., 2003; Fang et al., 2020). The calcium carbonate crystals bond the sand particles together and cause an improvement to the mechanical properties of MICP-treated sandy soils (Choi et al., 2020; Wen et al., 2020; Yuan et al., 2020; Cheng et al., 2021; Duan et al., 2021; Hu et al., 2021; Xue et al., 2021a; Xue et al., 2021b; Yan et al., 2021). The survivability of ureolytic bacteria may, however, be an issue when considered as *in situ* soil stabilizing measure. In contrast to MICP, EICP using purified urease has been considered as an alternative to MICP. Given that purified urease is commercially available, the legume, for example, can be a good alternative to commercial urease in calcium carbonate precipitation. It is not only urease-abundant but inexpensive and readily available (Namati and Voordouw, 2003; Yasuhara et al., 2012; Neupane et al., 2015a; Neupane et al., 2015b; Kavazanjian and Hamdan, 2015; Lee and Kim, 2020). Recent studies with a focus on the process of calcium carbonate crystallization have been conducted for the purpose of erosion resistance improvement (Sun et al., 2020; Ali and Karkush, 2021). Zango et al. (2021) found that the of leachate penetration pattern and hydraulic conductivities of the EICP-treated soils were considerably affected by the formation of biocementation or CaCO_3_ precipitation. Dakhane et al. (2018) indicated that the strength and fracture parameters (including fracture toughness and critical crack tip opening displacement) of mortars treated with an EICP solution scale well with the carbonate content. Arab et al. (2021) investigated the process of bio cementation using EICP and sodium alginate biopolymer to produce bio-bricks for use in the construction industry. The results show that the produced bio-bricks are comparable with cement-treated beams in terms of their mechanical properties and can also be considered an eco-friendly alternative to conventional bricks. Cui et al. (2020) illustrated that the unconfined compressive strength of sand treated using the one-phase low-pH method is much higher than that using the two-phase method, and the one-phase low-pH method is also simpler and more efficient as it involves a small number of injections. Furthermore, EICP technology offers significant potential for innovative and sustainable engineering applications in *in situ* field applications. Notwithstanding that, it is worthy to note that the successful application of EICP highly depends upon the interplay between the transport of urease as well as urea and calcium. In light of this, a comprehensive study on the mechanism of enzyme-induced carbonate precipitation and precipitation ratio and its influencing factors are in pressing need toward widening the horizon of application of EICP technology.

The enhancement mechanism of a few studies has been reported in which magnesium ions play a key role in elevating the carbonate precipitation during the EICP process (Putra et al., 2017a; Putra et al., 2017b; Xu et al., 2020; Putra et al., 2020; Wen et al., 2019b). Notwithstanding that, the biochemical reaction in relation to the EICP process is highly complex and can be affected by various factors. Putra et al. (2017a) indicated that adding magnesium chloride as a delaying agent reduces the reaction rate of the precipitation, which may further elevate the volume of the treated soil if applied to real cases due to the slower precipitation rate. Furthermore, magnesium chloride also enhances the amount of carbonate precipitation. Martin et al. (2021) indicated that the powdered milk leads to a stronger adhesion of the precipitated carbonate to surfaces while having minimal impact on the reaction kinetics. Cui et al. (2020) declared that the one-phase low-pH method can notably elevate the calcium conversion efficiency and the uniformity of calcium carbonate distribution in the sand samples when compared with the conventional two-phase EICP method. Almajed (2019) investigated the effect of adding biochar on the sand treated by a bio-inspired technique known as EICP and found that adding biochar decreases the cementation bonding between particles and subsequently the shear strength. It is worth noting that a small body of research start paying attention to their applications to the heavy metal immobilization of mine tailings (Li et al., 2013; Kang et al., 2016; Yang et al., 2016; Chen et al., 2018). As a result, studies with a focus on the mechanism affecting the carbonate precipitation are remarkably limited, while a large number of research pay attention to improving the shear strength of sandy soils using the EICP technology. The objectives of this study are 1) to conduct test tube experiments to reproduce the biomineralization process using the EICP technology and to document the carbonate precipitation, 2) to investigate the performance of carbonate precipitation of the modified EICP process under the effect of MgCl_2_, NH_4_Cl, and CaCl_2_ additions, respectively, toward revealing the regulation mechanisms, and 3) to explore the potential of applying the EICP technology to ancient structure protection.

## Materials and methods

### Materials and methods

**FIGURE 1:**
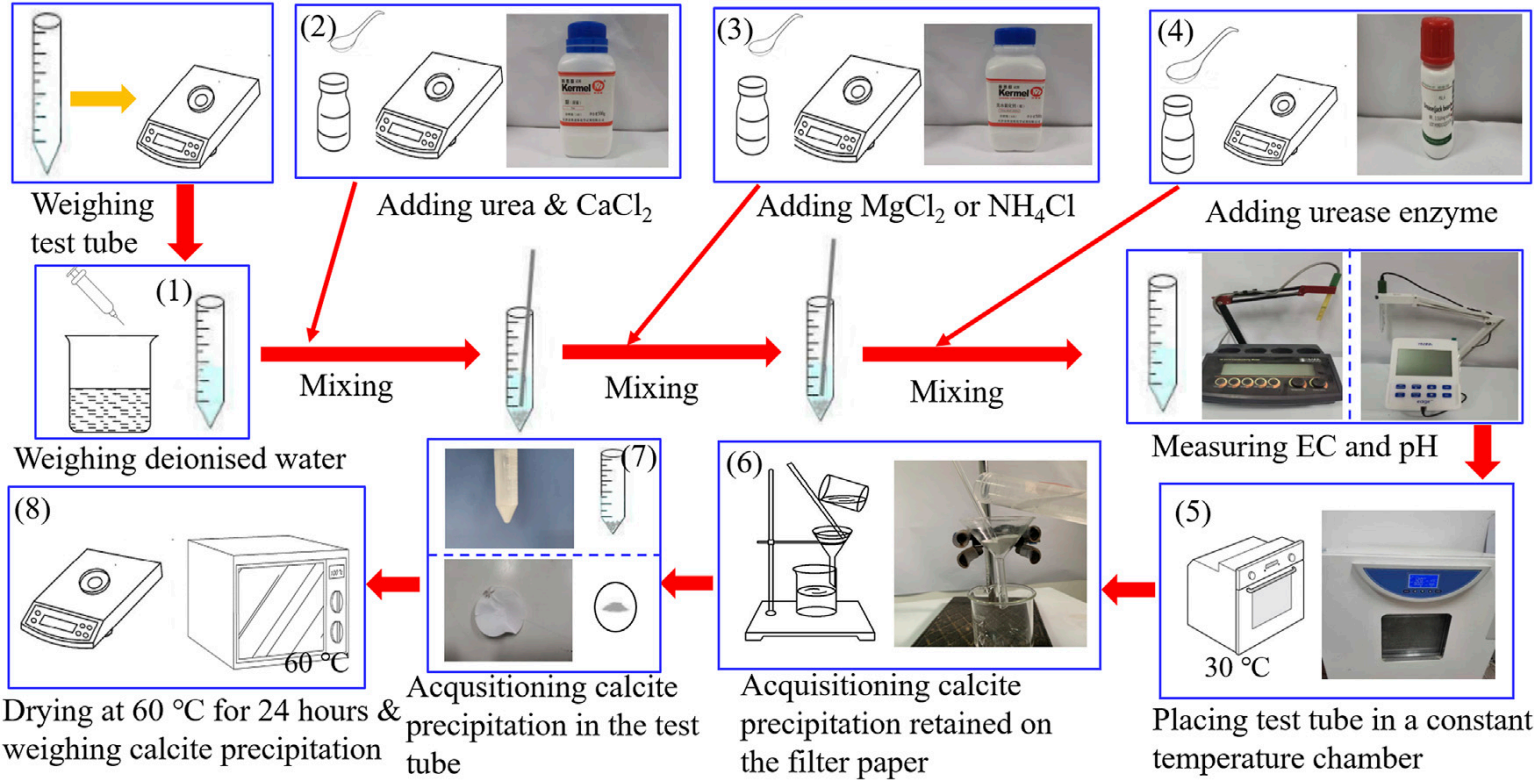
Schematic illustration of the test tube experiments.

Test tube experiments undertaken in this study utilized materials, including deionized water, 99% purity of urea, 96% purity of calcium chloride (CaCl_2_), and 98% purity of magnesium chloride (MgCl_2_) and ammonium chloride (NH_4_Cl). Furthermore, the activity of urease enzyme used to promote urea hydrolysis is 1.1 U/mg. CaCl_2_ was first poured into the prepared urea solution, followed by urea hydrolysis using the urease enzyme, finally producing the carbonate precipitation. This is referred to also as the ordinary EICP process, as shown in Figure 1. Furthermore, a modified EICP process was run in parallel, considering the effect of MgCl_2_ and NH_4_Cl and the concentration of CaCl_2_, as shown in Figure 1. The mass of CaCO_3_ precipitated in the ordinary and modified EICP processes was measured, and the precipitation ratio (PR) of CaCO_3_ was compared against each other to quantify the precipitation efficiency. PR can be calculated using:

$$\mathrm{PR}=\frac{\mathrm{Actual}\;\mathrm{precipitation}\;\mathrm{mass}}{\mathrm{Theoretical}\;\mathrm{precipitation}\;\mathrm{mass}}\times 100$$ (1)

where the theoretical precipitation mass is equal to *C* × *V* × *M*, in which *C* and *V* correspond to the concentration of urea–calcium carbonate solution in moles per liter and the total volume of EICP solution in liters respectively. *M* is the molar mass of calcium carbonate (100.087 g/mol) (Neupane et al., 2013; Putra et al., 2017b).

The calcite precipitation, resulting from the test tube experiments, was then applied to prevent ancient clay bricks from weathering erosion. Laboratory tests, including water absorption and air permeability tests, were conducted to verify the water-resisting properties of the EICP-treated clay bricks. Image binarization primarily aimed to evaluate the extent of the water-resistant film formed across the treated brick surface. The water absorption and air permeability of the EICP-treated bricks were defined using Eqs 2 and 3, respectively.

$$\frac{Q}{S}=K\sqrt{t}$$ (2)

$$T=\frac{M_{1}-M_{2}}{M_{1}}\times \mathrm{100}$$ (3)

where *Q* is equal to the water weight measured against different time intervals of immersion, *S* is the cross-section area of brick immersed in water, *K* is the slope in the Q/S-t^0.5^ diagram, also referred to as the sorptivity coefficient (g/cm^2^/s^0.5^), *t* is the duration for the water absorption tests, *M*_*1*_ is the mass of brick after the water saturation, *M*_*2*_ corresponds to the mass of brick after retrieval from the constant temperature chamber against different time intervals, and *T* corresponds to the percentage of weight loss (%).

### Test Tube Experiments

The test tube experiments primarily compare the carbonate precipitation of the ordinary EICP process with that of the modified EICP process when subjected to the effect of MgCl_2_, NH_4_Cl, and CaCl_2_ additions, respectively, and reveal the enhancement and degradation mechanisms of carbonate precipitation. In light of this, the test tube experiments consisted of one control group (A1 to A6 corresponding to the ordinary EICP process) and three test groups. One test group (B1 to B9 corresponding to the modified EICP process) was to investigate the effect of Mg addition on the performance of carbonate precipitation. The second test group (C1 to C6 corresponding to the modified EICP process) was principally to investigate the effect of NH_4_^+^ addition on the performance of carbonate precipitation. The third test group (D1 to D5 corresponding to the modified EICP process) mainly took into account the effect of CaCl_2_ addition. Measurements of pH and electric conductivity (EC) were accompanied with the test tube experiments, highlighting the relation of pH and EC with the performance of carbonate precipitation. The design of the test tube experiments is summarized in Table 1.

**TABLE 1:** Design of the test tube experiments.

Category		Urease enzyme	Urea	CaCl_2_	MgCl_2_	NH_4_Cl
		(kU/L)	(mol/L)	(mol/L)	(mol/L)	(mol/L)
Ordinary EICP	A1	5	0.125	0.125	—	—
	A2	5	0.25	0.25	—	—
	A3	5	0.50	0.50	—	—
	A4	5	0.75	0.75	—	—
	A5	5	1.00	1.00	—	—
	A6	5	1.25	1.25	—	—
Modified EICP under the effect of MgCl_2_ addition	B0	5	0.30	0.30	0.00	—
	B1	5	0.30	0.25	0.05	—
	B2	5	0.30	0.20	0.10	—
	B3	5	0.30	0.15	0.15	—
	B4	5	0.30	0.10	0.20	—
	C1	5	0.30	0.29	0.01	—
	C2	5	0.30	0.28	0.02	—
	C3	5	0.30	0.27	0.03	—
	C4	5	0.30	0.26	0.04	—
Modified EICP under the effect of NH_4_Cl addition	D1	5	0.30	0.30	—	0.05
	D2	5	0.30	0.30	—	0.10
	D3	5	0.30	0.30	—	0.15
	D4	5	0.30	0.30	—	0.20
	D5	5	0.30	0.30	—	0.30
Modified EICP under the effect of CaCl_2_ addition	E1	5	0.30	0.40	—	—
	E2	5	0.30	0.50	—	—
	E3	5	0.30	0.60	—	—
	E4	5	0.30	0.90	—	—

The flowchart of the test tube experiments is depicted in Figure 1. The procedure of test tube experiments is as follows: 1) The weight of the empty tube is first measured, and 25 ml of distilled water is added. 2) Urea and CaCl_2_ are added to the distilled water, 3) and a given proportion of MgCl_2_ or NH_4_Cl is added to the urea–CaCl_2_ solution. 4) Urease enzyme is the last to be added to the solution and stirred thoroughly to ensure the uniformity of the mixed solution. 5) The pH and EC of the mixed solution are measured during the urea hydrolysis process at a temperature of 30°C. 6) After that, the calcite precipitation is retained using a filter paper. 7) The calcite precipitation is also acquisitioned in the test tube, and 8) they are dried at 60°C for 24 h and weighed for its actual mass and precipitation ratio evaluation. The most popular methods for the measurement of urease activity include the Nessler’s reagent colorimetric method. The accuracy of the measured urease activity can be enhanced by a modified method proposed by van Paassen (2011) where electric conductivity (EC) measurements bring benefits to the enhancement of the accuracy. This is to say that EC can also be considered as a key indicator that reflects the urease activity. For this reason, the present work used the measurements of EC and pH to evaluate the urease activity and subsequently the degree of urea hydrolysis instead of direct measurement.

### Water Absorption Tests

Ancient brick specimens were dried at 110°C for 24 h and then placed in an incubator for cooling (30°C). Subsequently, their top surfaces were treated with an EICP solution of 25 ml for four times. The other surfaces, including bottom and peripheral surfaces, were thoroughly sealed with melting paraffin, thereby allowing a water seepage through the treated surface only. The brick specimens were then immersed in the deionized water, and their weights were measured at a given time interval. The sorptivity coefficient *K* was adopted as an indicator for assessing the water-resisting properties after the treatment (see Eq. 2). The higher the *K*, the poorer the water-resisting properties.

### Air Permeability Tests

Similarly, the top surface of the ancient brick specimens was treated with the EICP solution for four times. Prior to immersion in deionized water, they were weighed. They were then immersed in the deionized water to reach their saturation. The other surfaces of the brick specimens were sealed with melting paraffin right after their saturation. The *T* value was adopted to assess the weight loss against different time intervals when they were heated at 80°C (see Eq. 3). The lower air permeability can be attained using a smaller *T* value.

## Results and Discussion

### Ordinary Enzyme-Induced Carbonate Precipitation Process

**FIGURE 2:**
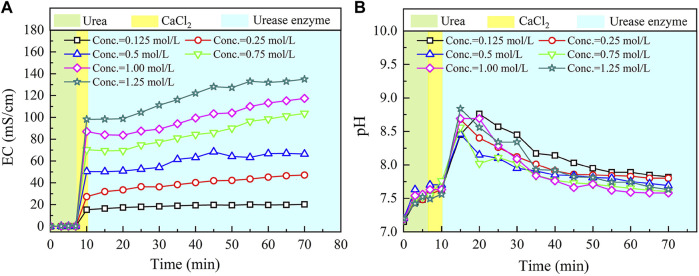
Temporal relations of electric conductivity (EC) and pH in the ordinary enzyme-induced carbonate precipitation (EICP) process: **(A)** EC and **(B)** pH.

The temporal relations of EC and pH in the ordinary EICP process when subjected to various concentrations of urea–CaCl_2_ are shown in Figure 2. EC shows a small change in the very beginning of the ordinary EICP process. This is not attributed to the ionization of urea but to the dissolution of urea. EC goes up very quickly after pouring CaCl_2_ into the urea solution because the ionization of CaCl_2_ produces a great number of ions that are capable of carrying electric charges. There are two chemical reactions initiated after pouring urease enzyme; these are urea hydrolysis, initiated by urease enzyme and produces NH_4_^+^ and CO_3_^2−^ ions (Eqs 4, 5), and carbonate precipitation, resulting from a chemical reaction between Ca^2+^ ions and CO_3_^2−^ ions, and consumes CO_3_^2−^ ions (Eq. 6).

$$CH_{4}N_{2}O+4H_{2}O\to H_{2}CO_{3}+2NH_{4}^{+}+2OH^{-}$$ (4)

$$\mathrm{HC}O_{3}^{-}+OH^{-}\to CO_{3}^{2-}+H_{2}O$$ (5)

$$Ca^{2+}+CO_{3}^{2-}\to \mathrm{CaC}O_{3}$$ (6)

The effect of the urea hydrolysis on EC outweighs the effect of carbonate precipitation, which is deemed as the main cause leading to a steadily increasing tendency of EC. EC consistently increases until the end of the ordinary EICP process. On the other hand, pH remains nearly unchanged after the introduction of CaCl_2_, but it shows a significant change when pouring urease enzyme into the urea–CaCl_2_ solution. This is due to the production of OH^−^ ions, induced by urea hydrolysis using urease enzyme. Then the pH goes into a decline until the end of the ordinary EICP process. The consumption of OH^−^ ions in the course of carbonate precipitation causes pH to decrease gently after reaching a peak value of 8.9.

**FIGURE 3:**
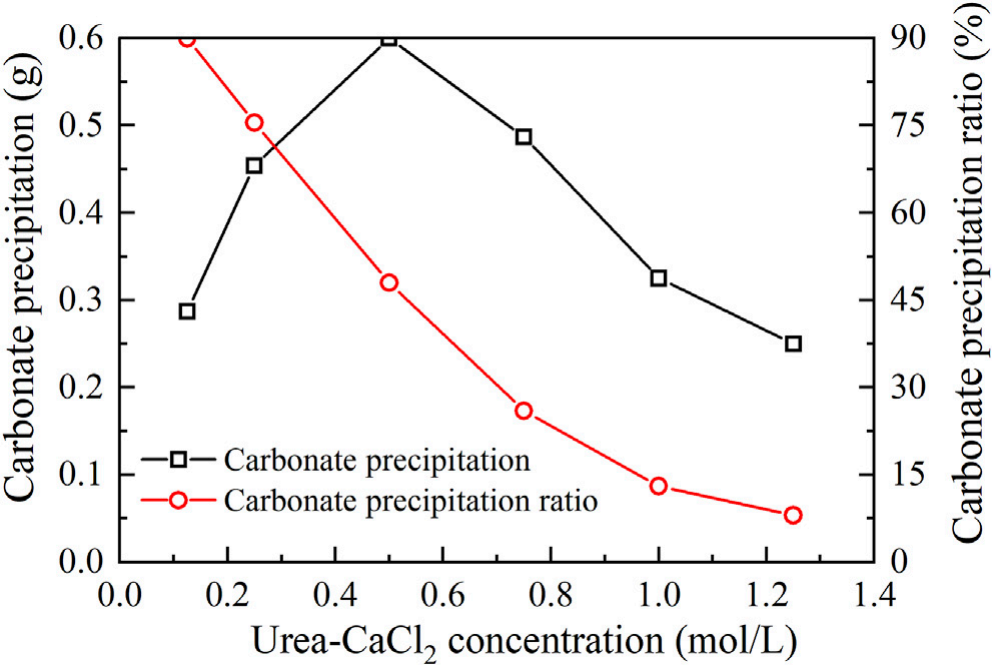
Relations of the actual mass of carbonate precipitation and the precipitation ratio (PR) versus the urea–CaCl_2_ concentration in the ordinary EICP process.

The relations of the actual mass of carbonate precipitation and the precipitation ratio (PR) versus the urea–CaCl_2_ concentration are shown in Figure 3. It is clear that the carbonate precipitation versus urea–CaCl_2_ concentration relation behaves in a “bell” shape, meaning that the highest carbonate precipitation cannot be achieved when subjected to higher or lower urea-CaCl_2_ concentrations. The lower urea-CaCl_2_ concentrations cannot provide enough basal constituents that are in great necessity of the EICP process toward causing a degradation of the carbonate precipitation, whereas for the higher urea–CaCl_2_ concentrations, the lower urease enzyme concentrations, in turn, impede the urea hydrolysis, leading to a degradation of the carbonate precipitation as well. In contrast, the PR versus urea–CaCl_2_ concentration relation presents a descending tendency all the way to the end of the EICP process, indicating that the lower the urea–CaCl_2_ concentration, the higher the PR. These results also indicate that it is either the highest carbonate precipitation or the highest PR, and we cannot have it both ways. Alternatively, a point where two curves intersect each other may provide a pathway to achieve both higher carbonate precipitation and PR. This point, for the ordinary EICP process, presents at the urea–CaCl_2_ concentration being equal to 0.3 mol/L.

### Effect of Magnesium Ion Addition

**FIGURE 4:**
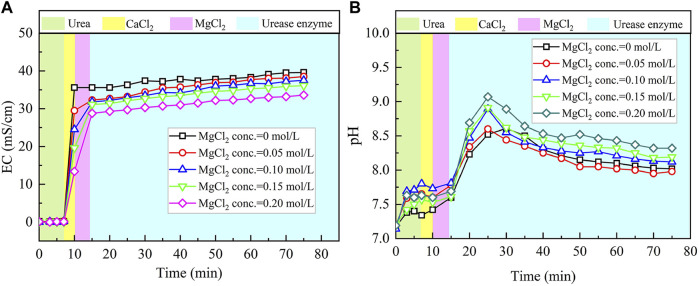
Temporal relations of EC and pH when subjected to the effect of MgCl_2_ addition: **(A)** EC and **(B)** pH.

**FIGURE 5:**
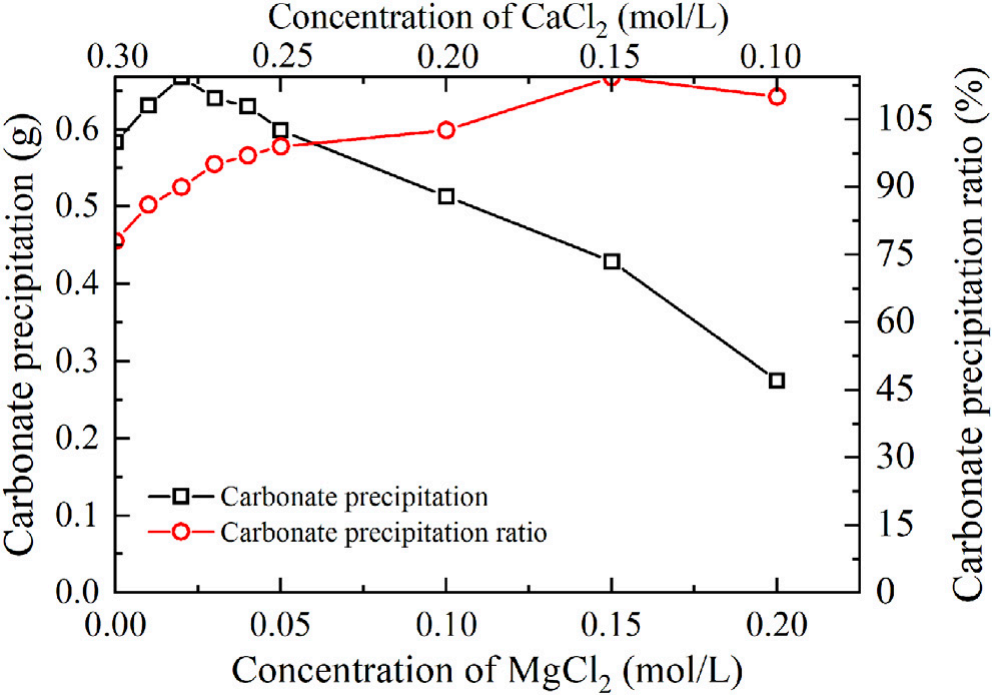
Relations of the actual mass of carbonate precipitation and the precipitation ratio (PR) versus the MgCl_2_ concentration in the modified EICP process.

The temporal relations of EC and pH when subjected to the effect of MgCl_2_ addition are shown in Figure 4. Given the higher carbonate precipitation and PR at 0.3 mol/L for the ordinary EICP process, the concentration of CaCl_2_ decreases from 0.3 to 0.1 mol/L, while the concentration of MgCl_2_ increases from 0 to 0.2 mol/L. This is to say that such an experimental design principally does not allow a total concentration in excess of 0.3 mol/L. When MgCl_2_ starts involving in the EICP process, the higher the concentration of MgCl_2_, the more significant the change in EC. However, compared with lower concentrations of MgCl_2_, higher concentrations of MgCl_2_ appear to cause some difficulty in elevating EC in the later stage of the modified EICP process. Similarly, pH shows a significant change when introducing urease enzyme to the urea-CaCl_2_ solution. The highest value being greater than 9 is higher than that of the ordinary EICP process. Subsequently, pH goes into a decline until the end of the modified EICP process. The relations of the carbonate precipitation and PR versus the concentration of MgCl_2_ are shown in Figure 5.

The carbonate precipitation for lower concentrations of MgCl_2_ (0–0.02 mol/L) increases with the increase in MgCl_2_ concentration, while for higher concentrations of MgCl_2_ (0.02–0.20 mol/L), it decreases with the increase in MgCl_2_ concentration. The highest calcite precipitation of 0.67 g for the modified EICP process under the effect of MgCl_2_ addition presents when the concentration of MgCl_2_ reaches 0.02 mol/L, which is higher than the highest calcite precipitation of 0.6 g for the ordinary EICP process (see Figures 3, 5). It is well known that the EICP process starts with urea hydrolysis toward discharging CO_3_^2−^. The carbonate precipitation is attained when these CO_3_^2−^ catch cations. The cations, including Ca^2+^ ions and Mg^2+^ ions, in solution are, however, bonded by water dipoles, and they have to be dehydrated prior to attracting CO_3_^2−^. Compared with Ca^2+^ ions, Mg^2+^ ions are more difficult to dehydrate because of its higher hydration enthalpy, thereby precipitating CaCO_3_ (calcite) crystals instead of MgCO_3_ (aragonite) crystals. Despite the difficulty in Mg^2+^ ion dehydration, the hydrated Mg^2+^ ions can poison the crystal surface. Mg^2+^ ions can only be bonded by two oxygen atoms resulting from two CO_3_^2-^ groups, which connection is relatively weaker than Ca^2+^ ions that are bonded by at least three oxygen atoms. For this reason, Mg^2+^ ions will be expelled by the following Ca^2+^ ions and cannot poison the growth of aragonite. Given a relatively higher solubility of magnesium calcite compared with pure calcite, the growth of calcite is inhibited. These results lead us to conclude that Mg^2+^ ions inhibit the growth of calcite and promotes aragonite precipitation.

The highest PR appears when the concentration of MgCl_2_ reaches 0.15 mol/L. To tackle the conflict, a point where two curves meet each other at a concentration of MgCl_2_ being about 0.05 mol/L prevents the degradation of carbonate precipitation and also secures the performance of PR. Furthermore, the lower carbonate precipitation, accompanied with higher concentrations of MgCl_2_, is observed. As discussed, although Mg^2+^ ions inhibit the growth of calcite, they promote another carbonate precipitation, namely, aragonite. Since aragonite is featured with smaller molecular weight compared with calcite, this causes the decreasing carbonate precipitation with the increasing concentration of MgCl_2_. On the other hand, it can be observed from Figures 3, 5 that Mg^2+^ ions cause the pH to reach its highest value 25 min after the beginning of the process; however, it reaches its highest value 15 min after the commencement of the process when Mg^2+^ ions do not take part in the process. This is to say that Mg^2+^ ions added to the urea-CaCl_2_ solution are difficult to dehydrate and delay the rate of urea hydrolysis, discharging OH^−^ ions at a slower rate. Notwithstanding that, Mg^2+^ ions promote the growth of aragonite in addition to calcite toward gearing up the performance of PR to higher than 114%.

### Effect of Ammonium Ion Addition

**FIGURE 6:**
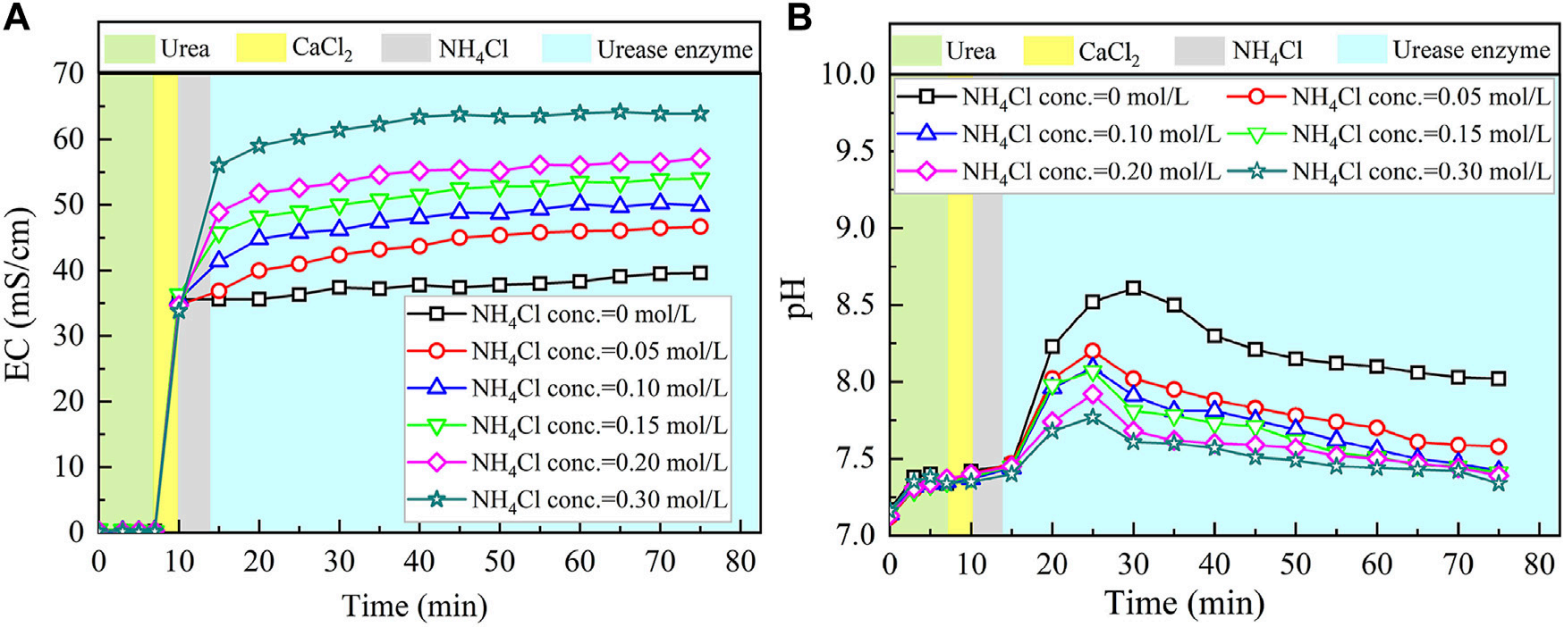
Temporal relations of EC and pH when subjected to the effect of NH_4_Cl addition: **(A)** EC and **(B)** pH.

The temporal relations of EC and pH when subjected to the effect of NH_4_Cl addition are shown in Figure 6. EC shows a rapid increase when NH_4_Cl takes part in the process. The higher the concentration of NH_4_Cl, the more significant the increase in EC. Higher concentrations of NH_4_Cl also result in higher levels of EC. Notwithstanding that, given that NH_4_Cl discharges NH_4_^+^ ions in the EICP process, the higher concentrations of NH_4_Cl, in turn, depress the urea hydrolysis (denoted by the blue area). Eq. 7 commences to move toward the opposite direction. EC, therefore, presents a small increase, followed by a negligible increase toward the end of the process.

$$CH_{4}N_{2}O+4H_{2}O\leftarrow H_{2}CO_{3}+2NH_{4}^{+}+2OH^{-}$$ (7)

**FIGURE 7:**
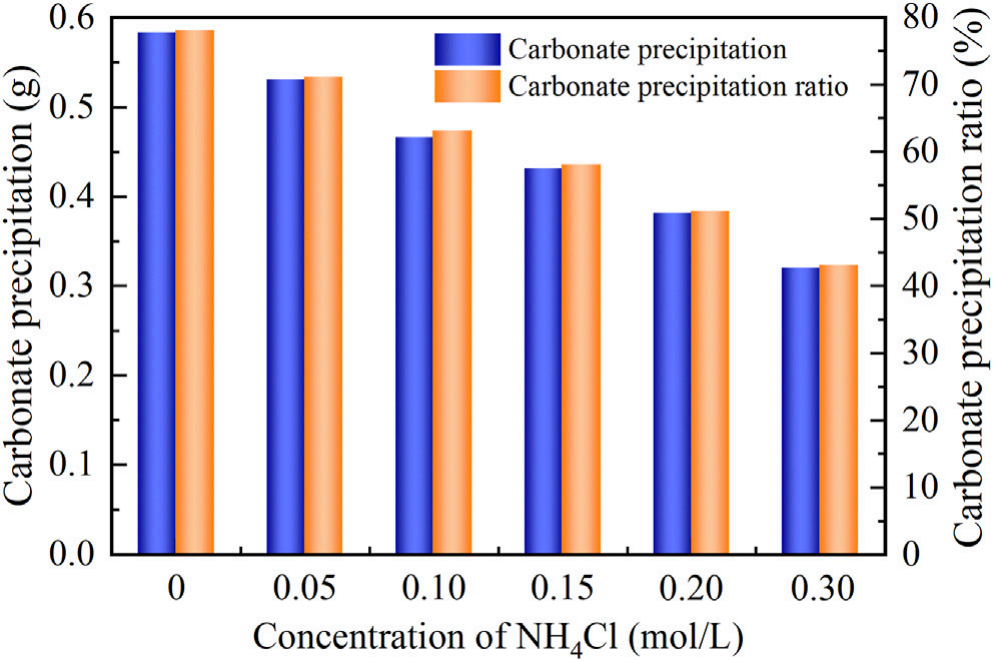
Relations of the actual mass of carbonate precipitation and the precipitation ratio (PR) versus the NH_4_Cl concentration in the modified EICP process.

As to the temporal relation of pH, NH_4_Cl added to the urea–CaCl_2_ solution do not cause pH to change notably (denoted by the gray area). The reverse EICP process causes a reduction in the discharge of OH^−^. In light of this, the highest value reduces to approximately 8.5 which is lower than 8.9 of the ordinary EICP process. The higher NH_4_Cl concentrations are deemed as the main cause leading to the decrease in pH. As a result, the higher the concentration of NH_4_Cl, the smaller the number of OH^−^ discharged, and the smaller the number of CO_3_^2−^, thereby degrading the carbonate precipitation. This also causes PR to monotonically decrease with the increase in the NH_4_Cl concentration, as shown in Figure 7.

### Effect of Calcium Chloride Addition

**FIGURE 8:**
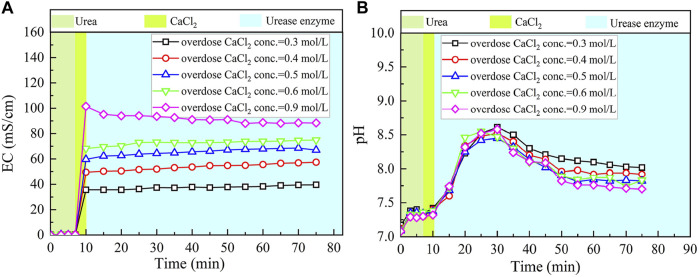
Temporal relations of EC and pH when subjected to the effect of CaCl_2_ addition: **(A)** EC and **(B)** pH.

**FIGURE 9:**
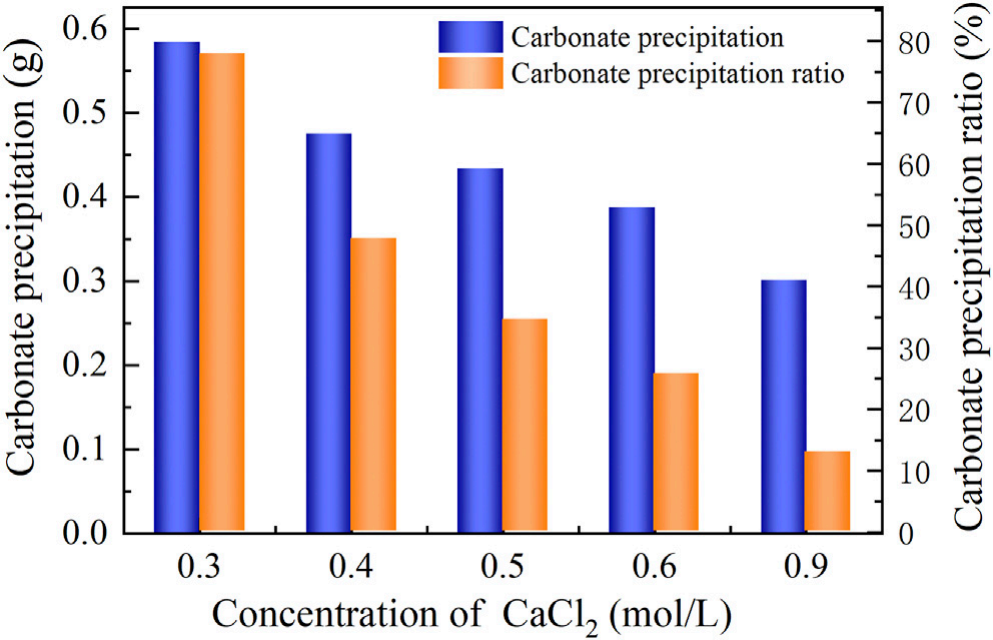
Relations of the actual mass of carbonate precipitation and the precipitation ratio (PR) versus the CaCl_2_ concentration in the modified EICP process.

The basal constituents applied to the process generally consisted of urea and CaCl_2_. To deepen our understanding of the effect of CaCl_2_ addition on the carbonate precipitation, test tube experiments, associated with higher concentrations of basal constituents, were conducted. The temporal relations of EC and pH under the effect of CaCl_2_ addition are depicted in Figures 8, 9. The higher the concentration of CaCl_2_, the more significant the change in EC. It can also be seen that the temporal relations of EC throughout the urea hydrolysis are relatively stable, indicating that the effect of carbonate precipitation counterbalances the effect of urea hydrolysis. In contrast, the concentration of CaCl_2_ appears to have a negligible effect on pH. Notwithstanding that, pH during the urea hydrolysis can also be characterized as a rapid increase and then a gentle decrease toward the end of the process. It can be regarded that the urea hydrolysis accompanies the discharge of OH^−^ toward the elevating pH very quickly. Then the carbonate precipitation commences to consume OH^−^, causing pH to go into a decline after reaching a peak. It is worth noting that the highest pH of 8.6, induced by the modified EICP process under the effect of CaCl_2_ addition is attained 30 min after the commencement of the process. Given that in the discharge of OH^−^ during the urea hydrolysis Ca(OH)_2_ will form indicates an occupation of OH^−^. The occupation of OH^−^ causes a lack of CO_3_^2−^, leading to a reduction in the carbonate precipitation. Furthermore, the occupation of OH^−^ also reduces pH, and for this reason, Ca(OH)_2_ that is just formed commences discharging OH^−^ to counterbalance the reduction of pH. This is considered as the main cause leading to a delay in the attendance of the highest pH (compared with the ordinary EICP process). Compared with PR of the modified EICP process under the effect of NH_4_Cl addition, the effect of CaCl_2_ addition degrades PR a step further by converting all Ca^2+^ ions into carbonate precipitation (see Eq. 1).

### Water Absorption and Air Permeability

**FIGURE 10:**
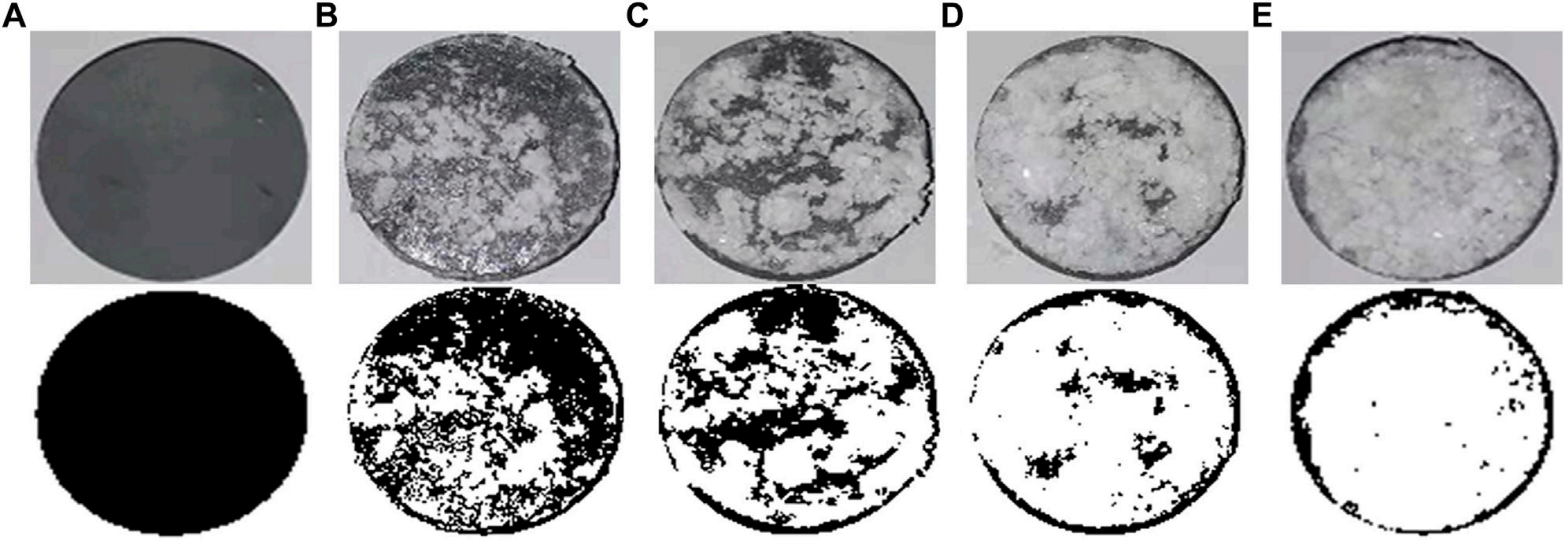
Brick specimen image binarization: **(A)** without EICP process, **(B)** with modified EICP process (once), **(C)** with modified EICP process (twice), **(D)** with modified EICP process (three times), and **(E)** with modified EICP process (four times).

**FIGURE 11:**
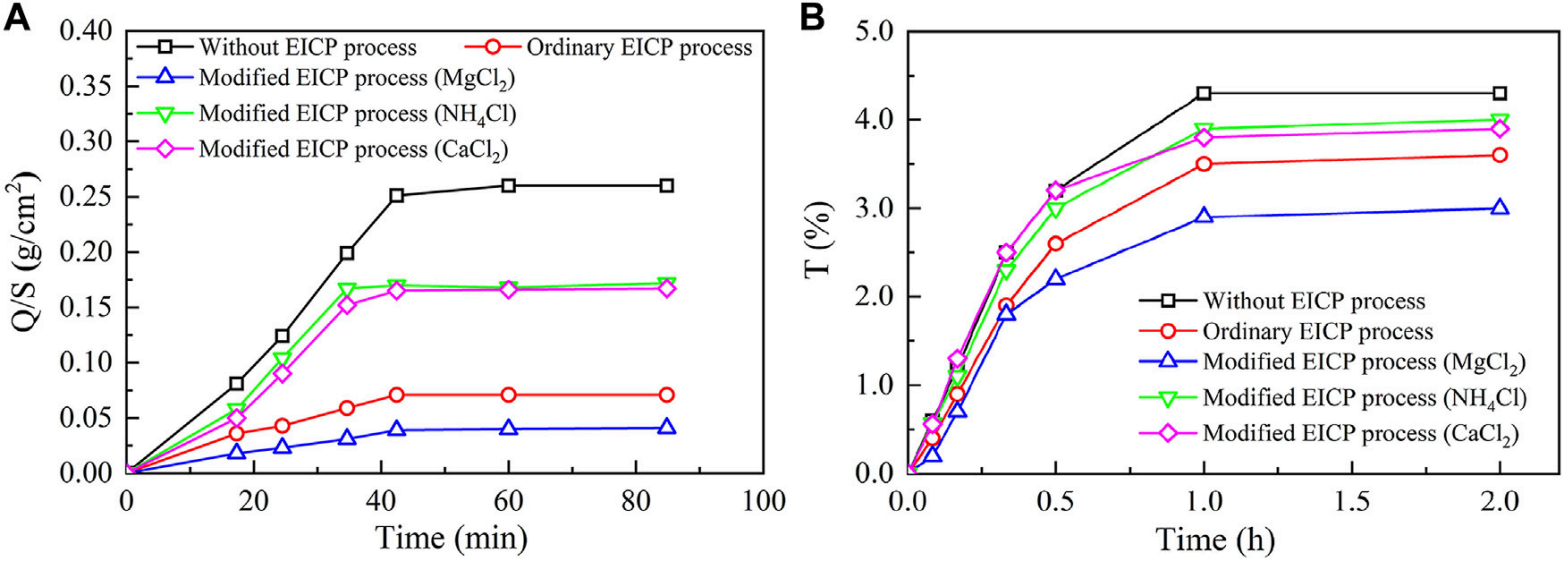
Water-resisting performance of the untreated, ordinary EICP treated, and modified EICP-treated brick specimens: **(A)** water absorption tests and **(B)** air permeability tests.

Figure 10 presents the image binarization results of the brick specimens without EICP process and with once, twice, three times, and four times of EICP process where the white color area indicates carbonate precipitation. The percentage extent of carbonate precipitation across the brick specimen surface elevates from 38.9% for once of the EICP process to 91.3% for four times of EICP process. The brick specimens with four times of EICP process were, therefore, applied to the subsequent water absorption and air permeability tests. The temporal relations of Q/S for the brick specimens subjected to the ordinary and modified EICP processes, respectively, are shown in Figure 11A. The Q/S increases sharply at the very beginning, and then the Q/S shows a little change 40 min after the commencement of the water absorption tests. The modified EICP-treated specimen subjected to the effect of higher MgCl_2_ concentrations is featured with the lowest water absorption. In contrast, the specimen without the EICP process is featured with the highest water absorption. Compared with the study of Liu et al. (2020b), their water adsorption is measured as 0.15 g/cm^2^ compared with 0.04 g/cm^2^ from the present work, although it may involve bacterial culture and inoculation. On the other hand, the temporal relations of T for the specimens subjected to the ordinary and modified EICP processes, respectively, are shown in Figure 11B. The modified EICP-treated specimen subjected to the effect of higher MgCl_2_ concentrations has the lowest mass loss corresponding to the lowest air permeability. The specimen without the EICP process has the highest mass loss, which also indicates the highest air permeability. These results not only represent the excellent water-resisting ability of the specimens but highlight the potential use of the EICP process for the protection of heritage buildings in NW China.

**FIGURE 12:**
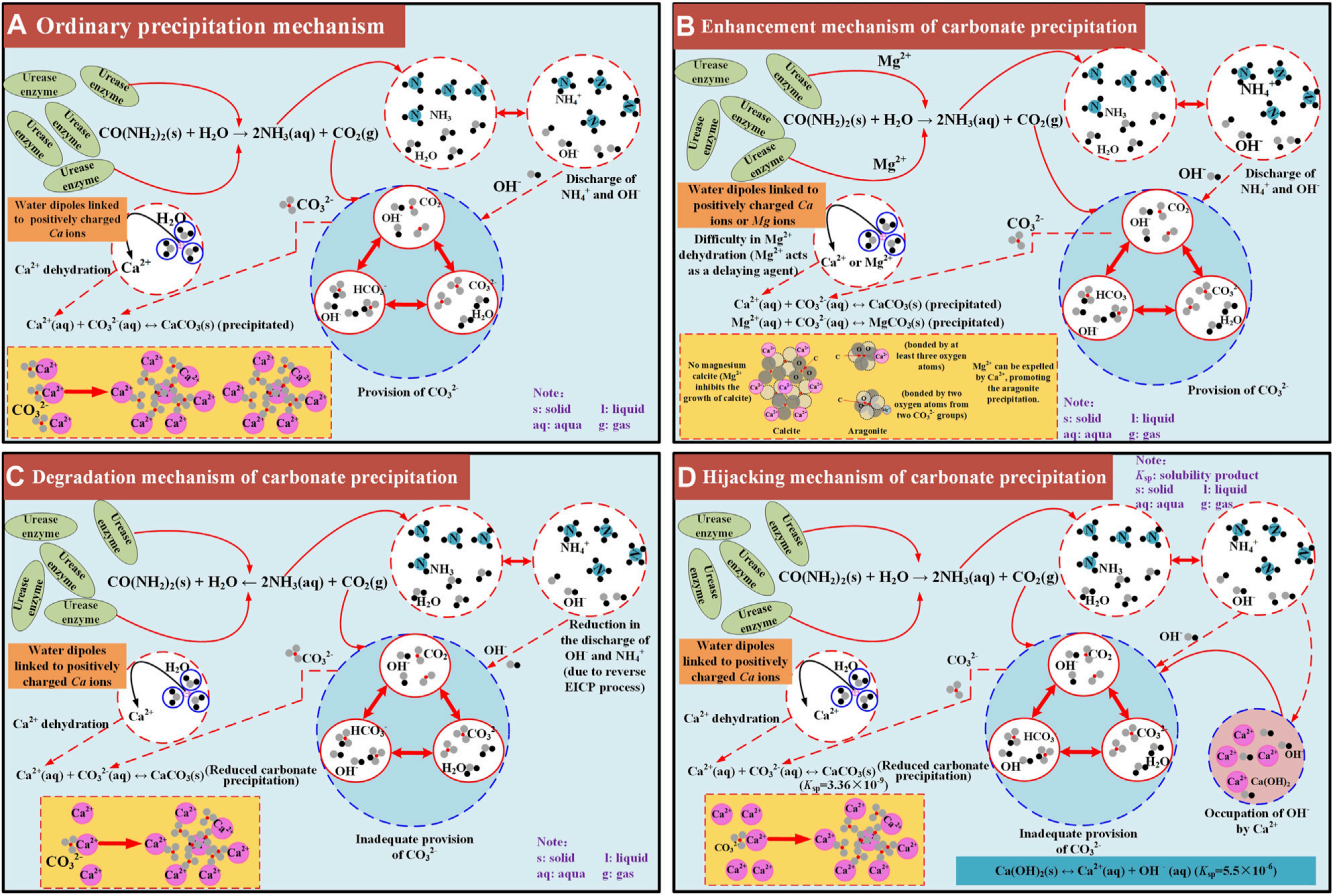
Schematic illustration of the carbonate precipitation mechanisms applied to the ordinary and modified EICP processes: **(A)** ordinary precipitation mechanism, **(B)** enhancement mechanism of carbonate precipitation, **(C)** degradation mechanism of carbonate precipitation, **(D)** hijacking mechanism of carbonate precipitation.

On the whole, the modified EICP process under the effect of MgCl_2_ addition performs the best, with the highest carbonate precipitation, followed by the ordinary EICP process (Figure 12A). In contrast, the modified EICP process when subjected to the effect of NH_4_Cl or CaCl_2_ addition performs the worst, with the lowest carbonate precipitation. Mg^2+^ ions added to the urea–CaCl_2_ solution delays the rate of urea hydrolysis due to the difficulty in their dehydration. Although Mg^2+^ ions inhibit the growth of calcite, they, however, favor aragonite precipitation toward producing more carbonate precipitation. These results reveal the enhancement mechanism of carbonate precipitation (see Figure 12B). The effect of NH_4_Cl addition not only reduces the discharge of OH^−^ by degrading the urease activity but also causes the EICP process to move toward the opposite direction. The lack of OH^−^ and reverse EICP process reduce the carbonate precipitation and reveal the degradation mechanism of carbonate precipitation (see Figure 12C). Last but not the least, the effect of CaCl_2_ addition leads to the occupation of OH^−^ and subsequently the lack of CO_3_^2−^, associated with the occupation of OH^−^, which causes a reduction in the carbonate precipitation, revealing the hijacking mechanism of carbonate precipitation (Figure 12D).

## Conclusion

This paper compared the carbonate precipitation of the ordinary EICP process to that of the modified EICP process under the effect of MgCl_2_, NH_4_Cl, and CaCl_2_ addition, respectively. The experimental results serve the purpose of interpreting the enhancement, degradation, and hijacking mechanisms of carbonate precipitation. Based on the results and discussion, some main conclusions can be drawn as follows:

a) The effect of magnesium ion addition delays the rate of urea hydrolysis because of the difficulty in its dehydration. Although the effect of magnesium ion addition inhibits the growth of calcite, it favors the aragonite precipitation, elevating the carbonate precipitation and carbonate precipitation ratio. These results further reveal the enhancement mechanism of carbonate precipitation.
b) The effect of ammonium ion addition, however, causes the enzyme-induced carbonate precipitation process to move toward the opposite direction, thereby reducing the concentration of carbonate ions, and subsequently, degrading the carbonate precipitation. Furthermore, the effect of calcium chloride addition leads to the occupation of hydroxide ions and the lack of carbonate ions toward degrading the carbonate precipitation. This is also referred to as the hijacking mechanism of carbonate precipitation.
c) The present work primarily aims to reveal the enhancement and degradation mechanisms affecting the carbonate precipitation throughout the enzyme-induced carbonate precipitation process. Further work in relation to field experiments are ongoing and would be discussed in another paper.

## Data Availability

The original contributions presented in the study are included in the article/Supplementary Material. Further inquiries can be directed to the corresponding author.

## References

[B1] Achal V. Mukherjee A. Reddy M. S. (2010). Microbial concrete: Way to Enhance the Durability of Building Structures. J. Mater. Civil Eng. 23 (6), 730–734. 10.1061/(ASCE)MT.1943-5533.0000159

[B2] Achal V. Mukerjee A. Sudhakara Reddy M. (2013). Biogenic Treatment Improves the Durability and Remediates the Cracks of concrete Structures. Construction Building Mater. 48, 1–5. 10.1016/j.conbuildmat.2013.06.061

[B3] Ali N. A. Karkush M. O. (2021). Improvement of Unconfined Compressive Strength of Soft Clay Using Microbial Calcite Precipitates. J. Eng. 27 (3), 67–75. 10.31026/j.eng.2021.03.05

[B4] Almajed A. Tirkolaei H. K. Eward K. J. (2018). Baseline Investigation on Enzyme-Induced Calcium Carbonate Precipitation. J. Geotechnical Geoenvironmental Eng. 144 (11), 04018081. 10.1061/(asce)gt.1943-5606.0001973

[B5] Almajed A. (2019). Enzyme Induced Cementation of Biochar-Intercalated Soil: Fabrication and Characterization. Arab J. Geosci. 12, 403. 10.1007/s12517-019-4557-z

[B6] Arab M. G. Omar M. Almajed A. Elbaz Y. Ahmed A. H. (2021). Hybrid Technique to Produce Bio-Bricks Using Enzyme-Induced Carbonate Precipitation (EICP) and Sodium Alginate Biopolymer. Construction Building Mater. 284, 122846. 10.1016/j.conbuildmat.2021.122846

[B48] Bai X.-D. Cheng W.-C. Li G. (2021a). A Comparative Study of Different Machine Learning Algorithms in Predicting EPB Shield Behaviour: a Case Study at the Xi'an Metro, China. Acta Geotech. 10.1007/s11440-021-01383-7

[B49] Bai X.-D. Cheng W.-C. Sheil B. B. Li G. (2021b). Pipejacking Clogging Detection in Soft Alluvial Deposits Using Machine Learning Algorithms. Tunnelling Underground Space Technology 113, 103908. 10.1016/j.tust.2021.103908

[B50] Bai X.-D. Cheng W. C. Dominic E. L. O. Li G. (2021c). Evaluation of Geological Conditions and Clogging of Tunneling Using Machine Learning. Geomechanics Eng. 25 (1), 59–73. 10.12989/gae.2021.25.1.059

[B7] Bang S. S. Galinat J. K. Ramakrishnan V. (2001). Calcite Precipitation Induced by Polyurethane-Immobilized Bacillus Pasteurii. Enzyme Microb. Technol. 28, 404–409. 10.1016/S0141-0229(00)00348-3 11240198

[B8] Bu C. Wen K. Liu S. Ogbonnaya U. Li L. (2018). Development of Bio-Cemented Constructional Materials through Microbial Induced Calcite Precipitation. Mater. Struct. 51 (1), 30. 10.1617/s11527-018-1157-4

[B9] Carmona J. P. S. F. Oliveira P. J. V. Lemos L. J. L. (2016). Biostabilization of a Sandy Soil Using Enzymatic Calcium Carbonate Precipitation. Proced. Eng. 143, 1301–1308. 10.1016/j.proeng.2016.06.144

[B10] Chen X. Guo H. Cheng X. (2018). Heavy Metal Immobilisation and Particle Cementation of Tailings by Biomineralisation. Environ. Geotechnics. 5 (2), 107–113. 10.1680/jenge.15.00068

[B52] Cheng W.-C. Duan Z. Xue Z.-F. Wang L. (2021). Sandbox Modelling of Interactions of Landslide Deposit with Terrace Sediments Aided by Field Observation. Bull. Eng. Geol. Environ. 80, 3711–3731. 10.1007/s10064-021-02144-2

[B11] Choi S.-G. Chang I. Lee M. Lee J.-H. Han J.-T. Kwon T.-H. (2020). Review on Geotechnical Engineering Properties of Sands Treated by Microbially Induced Calcium Carbonate Precipitation (MICP) and Biopolymers. Construction Building Mater. 246, 118415. 10.1016/j.conbuildmat.2020.118415

[B12] Cui M.-J. Lai H.-J. Hoang T. Chu J. (2020). One-Phase-Low-pH Enzyme Induced Carbonate Precipitation (EICP) Method for Soil Improvement. Acta Geotech. 16 (2), 481–489. 10.1007/s11440-020-01043-2

[B13] Dakhane A. Das S. Hansen H. O’Donnell S. Hanoon F. Rushton A. (2018). Crack Healing in Cementitious Mortars Using Enzyme-Induced Carbonate Precipitation: Quantification Based on Fracture Response. J. Mater. Civil Eng. 30 (4), 04018035. 10.1061/(asce)mt.1943-5533.0002218

[B53] Duan Z. Cheng W.-C. Peng J.-B. Rahman M. M. Tang H. (2021). Interactions of Landslide deposit with Terrace Sediments: Perspectives from Velocity of deposit Movement and Apparent Friction Angle. Eng. Geology. 280, 105913. 10.1016/j.enggeo.2020.105913

[B14] Fang X. Yang Y. Chen Z. Liu H. Xiao Y. Shen C. (2020). Influence of Fiber Content and Length on Engineering Properties of MICP-Treated Coral Sand. Geomicrobiology J. 37 (6), 582–594. 10.1080/01490451.2020.1743392

[B15] Hammes F. Boon N. de Villiers J. Verstraete W. Siciliano S. D. (2003). Strain-Specific Ureolytic Microbial Calcium Carbonate Precipitation. Appl. Environ. Microbiol. 69 (8), 4901–4909. 10.1128/AEM.69.8.4901-4909.2003 12902285 PMC169139

[B16] Hammes F. (2003). Ureolytic Microbial Calcium Carbonate Precipitation/door Frederik Hammes. Ph.D. thesis. Ghent: Ghent University. 10.1128/AEM.69.8.4901-4909.2003 PMC169139 12902285

[B17] Hu W. Cheng W.-C. Wen S. Mizanur Rahman M. (2021). Effects of Chemical Contamination on Microscale Structural Characteristics of Intact Loess and Resultant Macroscale Mechanical Properties. Catena 203, 105361. 10.1016/j.catena.2021.105361

[B18] Kang C.-H. Kwon Y.-J. So J.-S. (2016). Bioremediation of Heavy Metals by Using Bacterial Mixtures. Ecol. Eng. 89, 64–69. 10.1016/j.ecoleng.2016.01.023

[B19] Kavazanjian E. Hamdan N. (2015). “Enzyme Induced Carbonate Precipitation (EICP) Columns for Ground Improvement,” in IFCEE 2015. American Society of Civil Engineers, Reston, VA, USA, March 17–21, 2015, 2252–2261.

[B20] Lee S. Kim J. (2020). An Experimental Study on Enzymatic-Induced Carbonate Precipitation Using Yellow Soybeans for Soil Stabilization. KSCE J. Civ Eng. 24 (7), 2026–2037. 10.1007/s12205-020-1659-9

[B21] Li M. Cheng X. Guo H. (2013). Heavy Metal Removal by Biomineralization of Urease Producing Bacteria Isolated from Soil. Int. Biodeterioration Biodegradation 76, 81–85. 10.1016/j.ibiod.2012.06.016

[B22] Li M. Wen K. Li Y. Zhu L. (2017). Impact of Oxygen Availability on Microbially Induced Calcite Precipitation (MICP) Treatment. Geomicrobiology J. 35 (1-5), 15–22. 10.1080/01490451.2017.1303553

[B23] Li C. Bai S. Zhou T. Liu H. Qin X. Liu S. (2020). Strength-Increase Mechanism and Microstructural Characteristics of a Biotreated Geomaterial. Front. Struct. Civ. Eng. 14 (3), 599–608. 10.1007/s11709-020-0606-7

[B24] Liu S. Wang R. Yu J. Peng X. Cai Y. Tu B. (2020a). Effectiveness of the Anti-Erosion of an MICP Coating on the Surfaces of Ancient clay Roof Tiles. Construction Building Mater. 243, 118202. 10.1016/j.conbuildmat.2020.118202

[B25] Liu X. Koestler R. J. Warscheid T. Katayama Y. Gu J.-D. (2020b). Microbial Deterioration and Sustainable Conservation of Stone Monuments and Buildings. Nat. Sustain. 3 (12), 991–1004. 10.1038/s41893-020-00602-5

[B26] Martin K. Tirkolaei H. K. Kavazanjian E. (2021). Enhancing the Strength of Granular Material with a Modified Enzyme-Induced Carbonate Precipitation (EICP) Treatment Solution. Construction Building Mater. 271, 121529. 10.1016/j.conbuildmat.2020.121529

[B27] Namati M. Voordouw G. (2003). Modification of Porous media Permeability, Using Calcium Carbonate Produced Enzymatically *In Situ* . Enzyme Microb. Technol. 33 (5), 635–642. 10.1016/S0141-0229(03)00191-1

[B28] Neupane D. Yasuhara H. Kinoshita N. Unno T. (2013). Applicability of Enzymatic Calcium Carbonate Precipitation as a Soil-Strengthening Technique. J. Geotech. Geoenviron. Eng. 139 (12), 2201–2211. 10.1061/(asce)gt.1943-5606.0000959

[B29] Neupane D. Yasuhara H. Kinoshita N. Ando Y. (2015a). Distribution of Mineralized Carbonate and its Quantification Method in Enzyme Mediated Calcite Precipitation Technique. Soils and Foundations. 55 (2), 447–457. 10.1016/j.sandf.2015.02.018

[B30] Neupane D. Yasuhara H. Kinoshita N. Putra H. (2015b). Distribution of Grout Material within 1-m Sand Column in Insitu Calcite Precipitation Technique. Soils and Foundations. 55 (6), 1512–1518. 10.1016/j.sandf.2015.10.015

[B31] Putra H. Yasuhara H. Kinoshita N. Neupane D. Lu C.-W. (2016). Effect of Magnesium as Substitute Material in Enzyme-Mediated Calcite Precipitation for Soil-Improvement Technique. Front. Bioeng. Biotechnol. 4, 37. 10.3389/fbioe.2016.00037 27200343 PMC4854898

[B32] Putra H. Yasuhara H. Kinoshita N. (2017a). Optimum Condition for the Application of Enzyme-Mediated Calcite Precipitation Technique as Soil Improvement Technique. Int. J. Adv. Sci. Eng. Inf. Technol. 7 (6), 2145–2151. 10.18517/ijaseit.7.6.3425

[B33] Putra H. Yasuhara H. Kinoshita N. Hirata A. (2017b). Optimization of Enzyme-Mediated Calcite Precipitation as a Soil-Improvement Technique: The Effect of Aragonite and Gypsum on the Mechanical Properties of Treated Sand. Crystals. 7 (2), 59. 10.3390/cryst7020059

[B34] Putra H. Yasuhara H. Erizal S. Fauzan M. (2020). Review of Enzyme-Induced Calcite Precipitation as a Ground-Improvement Technique. Infrastructures. 5 (8), 66. 10.3390/infrastructures5080066

[B35] Sun X. Miao L. Yuan J. Wang H. Wu L. (2020). Application of Enzymatic Calcification for Dust Control and Rainfall Erosion Resistance Improvement. Sci. Total Environ. 759 (14), 143468. 10.1016/j.scitotenv.2020.143468 33277016

[B36] Sun X. Miao L. Wang H. Yuan J. Fan G. (2021). Enhanced Rainfall Erosion Durability of Enzymatically Induced Carbonate Precipitation for Dust Control. Sci. Total Environ. 791 (29), 148369. 10.1016/j.scitotenv.2021.148369 34126498

[B37] Tittelboom K. V. N. Belie D. Muynck W. D. Verstraete W. (2010). Use of Bacteria to Repair Cracks in Concrete. Cement Concrete Res. 40 (1), 157–166. 10.1016/j.cemconres.2009.08.025

[B38] van Paassen L. A. (2011). “Bio-Mediated Ground Improvement: from Laboratory experiment to Pilot Applications,” in Geo-Frontiers Congress 2011, Dallas, Texas, United States, March 13-16, 2011 (ASCE), 4099–4108. 10.1061/41165(397)419

[B39] Wen K. Li Y. Liu S. Bu C. Li L. (2019a). Development of an Improved Immersing Method to Enhance Microbial Induced Calcite Precipitation Treated sandy Soil through Multiple Treatments in Low Cementation media Concentration. Geotech Geol. Eng. 37 (2), 1015–1027. 10.1007/s10706-018-0669-6

[B40] Wen K. Li Y. Liu S. Bu C. Li L. (2019b). Evaluation of MICP Treatment through EC and pH Tests in Urea Hydrolysis Process. Environ. Geotechnics 8, 274–281. 10.1680/jenge.17.00108

[B41] Wen K. Li Y. Amini F. Li L. (2020). Impact of Bacteria and Urease Concentration on Precipitation Kinetics and Crystal Morphology of Calcium Carbonate. Acta Geotech. 15 (1), 17–27. 10.1007/s11440-019-00899-3

[B51] Wu A. Cheng W.-C. Kang N. Shang S. Xiao W. Yuan K. (2021). Internal Erosion Behaviour of Compacted Loess Against Different Hydraulic Conditions Indicated by Enhanced Pinhole Tests. Arab J. Geosci. 14, 2178. 10.1007/s12517-021-08583-1

[B42] Xiao Y. Ma G. L. Nan B. W. McCartney J. S. (2020). Thermal Conductivity of Granular Soil Mixtures with Contrasting Particle Shapes. J. Geotechnical Geoenvironmental Eng. 146 (5), 06020004. 10.1061/(asce)gt.1943-5606.0002243

[B43] Xu X. Guo H. Cheng X. Li M. (2020). The Promotion of Magnesium Ions on Aragonite Precipitation in MICP Process. Construction Building Mater. 263 (3-4), 120057. 10.1016/j.conbuildmat.2020.120057

[B54] Xue Z.-F. Cheng W.-C. Wang L. (2021a). Effect of Straw Reinforcement on the Shearing and Creep Behaviours of Quaternary Loess. Sci. Rep. 11, 19926. 10.1038/s41598-021-99318-5 PMC8497581 34620918

[B55] Xue Z.-F. Cheng W.-C. Wang L. Song G. (2021b). Improvement of the Shearing Behaviour of Loess Using Recycled Straw Fiber Reinforcement. KSCE J. Civ Eng. 25 (9), 3319–3335. 10.1007/s12205-021-2263-3

[B56] Yan X. Duan Z. Sun Q. (2021). Influences of Water and Salt Contents on the thermal Conductivity of Loess. Environ. Earth Sci. 80, 52. 10.1007/s12665-020-09335-2

[B44] Yang J. Pan X. Zhao C. Mou S. Achal V. Al-Misned F. A. (2016). Bioimmobilization of Heavy Metals in Acidic Copper Mine Tailings Soil. Geomicrobiology J. 33 (3-4), 261–266. 10.1080/01490451.2015.1068889

[B45] Yasuhara H. Neupane D. Hayashi K. Okamura M. (2012). Experiments and Predictions of Physical Properties of Sand Cemented by Enzymatically-Induced Carbonate Precipitation. Soils and Foundations 52 (3), 539–549. 10.1016/j.sandf.2012.05.011

[B46] Yuan H. Ren G. Liu K. Zheng W. Zhao Z. (2020). Experimental Study of EICP Combined with Organic Materials for Silt Improvement in the Yellow River Flood Area. Appl. Sci. 10, 7678. 10.3390/app10217678

[B47] Zango M. U. Kassim K. A. Sa’ari R. Rashid M. F. A. Muhammed A. S. Ahmad K. (2021). Use of Digital Image Technique to Study Leachate Penetration in Biocemented Residual Soil. Mater. Today Proc. 2, 1–7. 10.1016/j.matpr.2021.02.211

